# Association between ferritin to albumin ratio and 28-day mortality in patients with sepsis: a retrospective cohort study

**DOI:** 10.1186/s40001-023-01405-y

**Published:** 2023-10-10

**Authors:** Feng Liu, Zhengting Liu

**Affiliations:** 1 Ganzhou Maternal and Child Care Service Center, Ganzhou, Jiangxi China; 2 Department of Clinical Laboratory, The Affiliated Ganzhou Hospital of Nanchang University, Ganzhou, Jiangxi China

**Keywords:** Sepsis, Ferritin, Albumin, FAR, MIMIC-IV, 28-Day mortality

## Abstract

**Objectives:**

The ratio of ferritin to albumin (FAR) has been proposed as a novel prognostic indicator for COVID-19. However, the role of FAR in predicting the all-cause mortality rate in patients with sepsis has not been evaluated. Therefore, the aim of this study is to elucidate the correlation between FAR and the 28-day all-cause mortality rate in patients with sepsis.

**Methods:**

This study used data from the Medical Information Mart for Intensive Care IV database (v2.0) for a retrospective cohort analysis. The study focused on adult patients with sepsis who were admitted to the intensive care unit. The primary objective was to assess the predictive capability of FAR in determining the 28-day all-cause mortality rate among patients with sepsis.

**Results:**

The study involved 1553 sepsis patients in total. Based on the survival status of sepsis patients within 28 days, they were divided into two groups: a survival group consisting of 973 patients, and a death group consisting of 580 patients. The results revealed a 28-day mortality rate of 37.35% among sepsis patients. The multivariable Cox regression analysis revealed that FAR was an independent predictor of the 28-day all-cause mortality rate in patients with sepsis (hazard ratio [HR]: 1.17–1.19; 95% confidence interval 1.11–1.26; *P* < 0.001). The FAR demonstrated a higher area under the curve (AUC) of 61.01% (95% confidence interval 58.07–63.96%), compared to serum ferritin (60.48%), serum albumin (55.56%), and SOFA score (56.97%). Receiver operating characteristic curve (ROC) analysis determined the optimal cutoff value for FAR as 364.2215. Kaplan–Meier analysis revealed a significant difference in the 28-day all-cause mortality rate between patients with FAR ≥ 364.2215 and those with FAR < 364.2215 (*P* < 0.001). Furthermore, subgroup analysis showed no significant interaction between FAR and each subgroup.

**Conclusions:**

This study revealed a significant correlation between FAR and the 28-day mortality rate in patients with sepsis. Higher FAR values were strongly associated with increased mortality rates within 28 days.

## Introduction

Sepsis is described as a severe infectious disease characterized by an abnormal inflammatory response of the body to an infection, leading to tissue damage and organ dysfunction [1]. Despite advancements in sepsis research in recent years, it remains a significant cause of death among hospitalized patients, imposing a tremendous economic burden on patients' families [2]. Recent studies have reported alarming mortality rates for sepsis in the United States, with the annual rate among hospitalized patients as high as 26.7%, and reaching a staggering 41.9% in the intensive care unit [3]. Therefore, early detection and treatment of sepsis are crucial for improving patients' prognosis. Currently, we use scoring tools such as qSOFA, SOFA, and APACHE to assess the severity of sepsis in patients [4]. However, these scores require the collection of multiple indicators, making their clinical application less convenient. Consequently, there is an urgent need to identify a simple, fast, and cost-effective predictive indicator to evaluate the severity of sepsis.

Ferritin is a widely present protein in plasma, cells, and tissue organs [5]. Studies indicate its crucial role in infection and inflammation processes [6, 7]. As a result, serum ferritin level is often considered a significant indicator for infectious and inflammatory diseases. An increase in ferritin levels suggests the presence of inflammation or the severity of a disease. Moreover, research has found associations between abnormal serum ferritin levels and conditions, such as digestive disorders [8], Liver disease [9, 10] and COVID-19 [11]. However, it is worth noting that ferritin levels are influenced by various factors, including inflammation, liver function, and nutritional status. Therefore, relying solely on ferritin levels may not provide reliable results. On the other hand, serum albumin, a negative acute-phase reactant protein, is closely linked to the degree of inflammation, disease prognosis, and mortality rates [12]. Current studies demonstrate a strong correlation between serum albumin and mortality and prognosis in conditions, such as sepsis [13], tumors [14], and kidney diseases [15]. It is important to consider that patients' nutritional status can also impact albumin levels. Therefore, using albumin levels alone to predict disease prognosis may have certain limitations.

Currently, there have been studies indicating the association between ferritin/albumin and the prognosis of COVID-19 patients [16]. Nevertheless, the relationship between ferritin/albumin levels and mortality rates in patients with sepsis is still not clear. Therefore, we assessed the relationship between ferritin/albumin and the 28-day all-cause mortality rate in patients with sepsis based on the MIMIC-IV database. In accordance with the STROBE guidelines, we present the following article.

## Methods

### Database introduction

The data for this study was obtained from the MIMIC-IV (v2.0) database, a publicly available database for medical research which contains inpatient data from Beth Israel Deaconess Medical Center in Boston, USA, from 2008 to 2019. MIMIC-IV 2.0 contains various types of patient information, including demographic data, physiological characteristics, diagnoses, surgeries, medication prescriptions, laboratory test results, and vital signs. To gain access to and make effective use of the database for this study, Zhengting Liu, the corresponding author, successfully completed the Collaborative Institutional Training Initiative (CITI) program, specifically passing exams on "Conflicts of Interest" and "Data or Specimen Only Research" (ID: 48255890). Consequently, the research team is now authorized and eligible to access and extract the necessary data from the database for their study.

### Population selection criteria

**Fig. 1:**
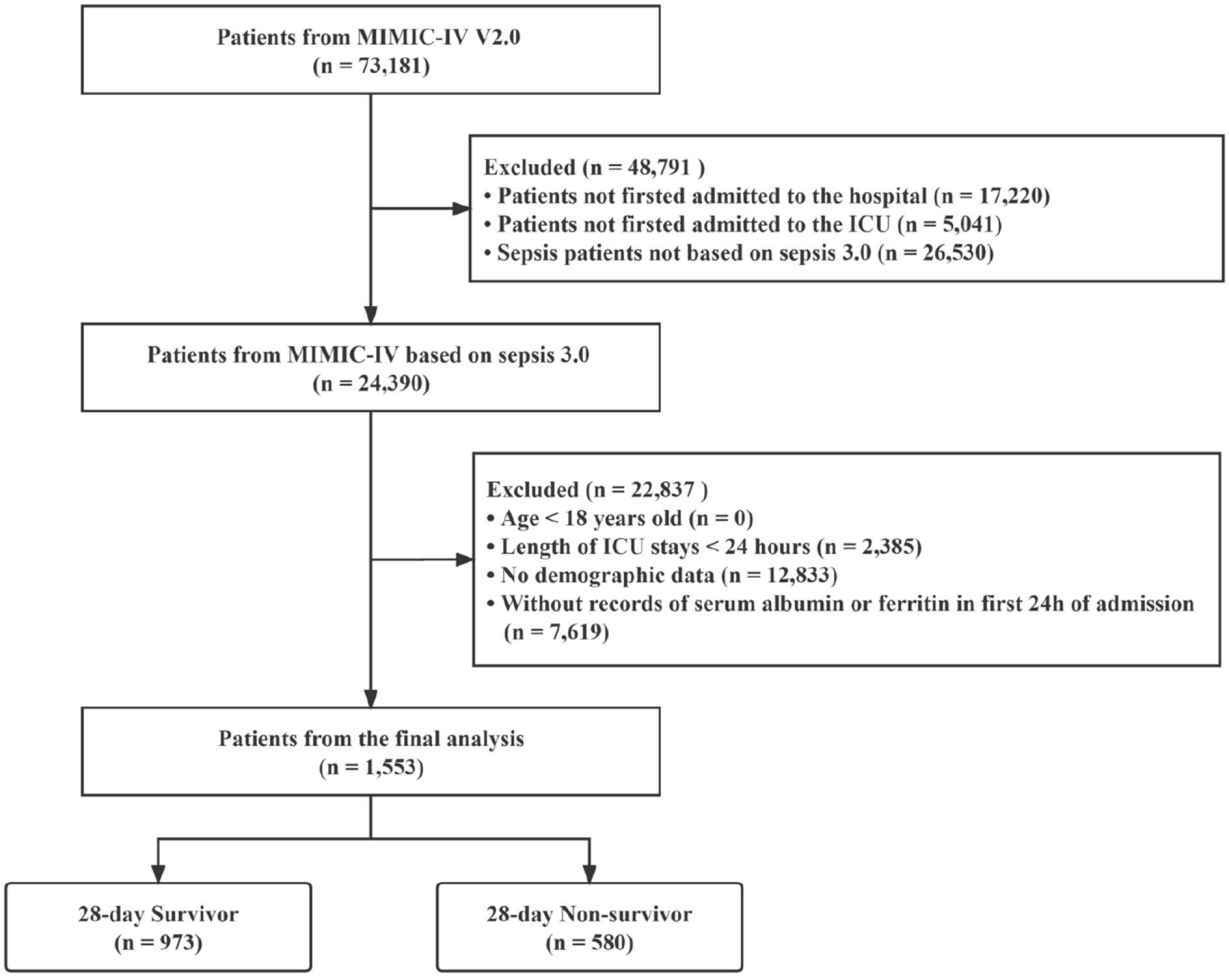
Flowchart of study patients

According to the MIMIC-IV database, a total of 73,181 patients were admitted to the ICU between 2008 and 2019, of which 50,920 were first-time hospitalizations and first-time ICU stays. Among them, 24,390 met the definition of Sepsis-3, which is defined as an increase of ≥ 2 points in Sequential Organ Failure Assessment (SOFA) score plus documented or suspected infection. After a thorough screening process, patients who fail to meet the following criteria will be excluded: (1) patients who are under the age of 18; (2) patients who have had less than 24 h of stay in the ICU; (3) patients with missing death time data; and (4) patients who are not recorded for serum ferritin and albumin within 24 h of hospitalization. This study ultimately included a cohort of 1553 patients (Fig. 1).

### Data extraction

The FAR is the primary variable under investigation. To reduce the influence of subsequent treatments on serum ferritin and albumin, we opted to measure these factors for the first time after patients were admitted. Furthermore, we identified and controlled for several other potential confounding factors, including demographic variables (age and gender), vital signs (heart rate, respiratory rate, systolic and diastolic blood pressure, mean arterial pressure, temperature, and oxygen saturation), laboratory indicators (albumin, ferritin, anion gap, bicarbonate, calcium, chloride, sodium, potassium, hematocrit, hemoglobin, red blood cells, erythrocyte distribution width, glucose, blood urea nitrogen, creatinine, platelets, and white blood cells), comorbidities (myocardial infarction, congestive heart failure, chronic pulmonary disease, diabetes, renal disease, malignant cancer, severe liver disease, and metastatic solid tumor), and sequential organ failure assessment (SOFA). Data extraction was performed using structured query language (SQL) queries run on PostgresSQL software (v13.7.1) and Navicat Premium software (version 15) 0.2.4 Grouping and endpoint events.

### Grouping and endpoint events

In this study, patients were stratified into two groups: a 28-day survival group and a 28-day death group, based on their mortality status within the specified timeframe. The primary endpoint of the study was defined as mortality from any cause within 28 days.

### Management of missing data

To minimize bias, any variable with missing data greater than 15% (such as aspartate aminotransferase, lactate dehydrogenase, lactate, C-reactive protein, and coagulation indicators) was excluded from the study. For variables with less than 5% missing data, including mean arterial pressure, heart rate, oxygen saturation, temperature, calcium ion, hematocrit, hemoglobin, and red blood cells, the missing values were replaced with the mean value of the variable. Platelets and white blood cells had less than 5% missing data and were replaced with the median value of the variable.

### Statistical analysis

In this study, normally distributed continuous variables were presented as mean ± standard deviation, while non-normally distributed continuous variables were represented using the median or interquartile range. Categorical variables, on the other hand, were expressed as percentages. To analyze the baseline characteristics, *t* tests or one-way analysis of variance (ANOVA) were used for continuous variables, while Chi-square tests were employed for categorical variables. Univariate COX regression analysis was conducted to identify potential risk factors for 28-day mortality in sepsis patients, specifically considering variables with a multivariate COX regression analysis *P* value of less than 0.001. Moreover, ROC curve analysis was carried out to evaluate and compare the prognostic performance of serum ferritin, serum albumin, the ferritin/albumin ratio (FAR), and Sequential Organ Failure Assessment (SOFA) score in predicting 28-day mortality among sepsis patients. Sensitivity and specificity measures were computed for each indicator. The Youden index was used to determine the optimal cutoff value for FAR, and patients were separated into high and low FAR groups for survival rate comparisons using Kaplan–Meier survival analysis. Finally, subgroup analysis was performed to determine if an interaction effect of FAR existed in different subgroups. Statistical analyses were conducted using R 4.1.1 software and Freestat software version 1.8. The significance level was set at *P* < 0.05 for all tests, using a two-tailed approach.

## Results

### Baseline patient demographics

Table 1 displays the baseline characteristics of sepsis patients in both the 28-day survival and non-survival groups. This study included a total of 1553 patients who met the inclusion and exclusion criteria, consisting of 687 females and 866 males, with an average age of 66.8 ± 15.1 years. The 28-day mortality rate for sepsis patients was 37.34%. Non-survivors had significantly lower hospital length of stay, SBP, DBP, MAP, and SpO2, and higher RR and SOFA scores than survivors (*P* < 0.05). In addition, laboratory indices such as FAR, ferritin, AG, RDW, BUN, CREA, and WBC were higher in the non-survival group, whereas ALB, bicarbonate, chloride, and PLT were lower (*P* < 0.05). No statistically significant differences were found between the two groups for the remaining covariates (*P* > 0.05). Among the sepsis patients, 28.3%, 20.7%, 13.1%, and 8.8% had nephropathy, severe liver disease, tumor, and diabetes mellitus, respectively.

**Table 1:** Baseline characteristics between survivors and non-survivors

Variables	Total (*n* = 1553)	Survivors (*n* = 973)	Non survivors (*n* = 580)	*P*
Age (years)	66.8 ± 15.1	66.9 ± 14.5	66.7 ± 16.0	0.823
Gender (%)				0.868
Female	687 (44.2)	432 (44.4)	255 (44)	
Male	866 (55.8)	541 (55.6)	325 (56)	
Length of stay in hospital	349.8 (190.3, 633.8)	475.9 (242.4, 798.3)	234.2 (122.1, 374.0)	< 0.001
Length of stay in ICU	135.6 (70.6, 286.1)	136.1 (69.8, 328.1)	131.2 (73.9, 244.0)	0.064
Vital signs				
HR (beats/min)	94.9 ± 22.6	94.1 ± 22.5	96.2 ± 22.8	0.084
SBP (mmHg)	119.2 ± 24.6	120.2 ± 25.2	117.4 ± 23.5	0.026
DBP (mmHg)	65.4 ± 18.3	66.2 ± 18.8	64.0 ± 17.2	0.023
MBP (mmHg)	79.3 ± 18.9	80.3 ± 19.2	77.6 ± 18.2	0.006
RR (t/min)	21.1 ± 6.3	20.8 ± 6.1	21.6 ± 6.6	0.017
SpO_2_ (%)	96.2 ± 4.9	96.5 ± 4.5	95.8 ± 5.3	0.006
Temperature (℃)	36.7 ± 1.0	36.7 ± 1.0	36.6 ± 1.1	0.068
Laboratory parameters				
ALB (g/dL)	2.9 ± 0.7	2.9 ± 0.7	2.8 ± 0.7	< 0.001
Ferritin (ng/ml)	532.0 (230.0, 1199.0)	467.0 (209.0, 1011.0)	777.0 (292.8, 1847.8)	< 0.001
FAR	188.7 (78.5, 462.9)	164.2 (69.4, 355.9)	292.9 (107.3, 669.5)	< 0.001
AG (mg/dL)	17.7 ± 6.0	17.1 ± 5.6	18.8 ± 6.4	< 0.001
Bicarbonate (mg/dL)	21.4 ± 6.0	21.8 ± 6.0	20.8 ± 6.0	0.002
Calcium (mg/dL)	8.2 ± 1.2	8.2 ± 1.2	8.2 ± 1.1	0.749
Chloride(mEq/L)	101.8 ± 8.5	102.2 ± 8.5	101.2 ± 8.5	0.026
Sodium (mEq/L)	137.4 ± 7.4	137.4 ± 7.4	137.4 ± 7.4	0.929
Potassium (mEq/L)	4.4 ± 1.0	4.4 ± 1.0	4.5 ± 1.0	0.472
HCT (%)	31.2 ± 7.1	31.4 ± 7.1	31.0 ± 7.0	0.351
HGB (g/dL)	10.1 ± 2.3	10.2 ± 2.3	10.1 ± 2.4	0.388
RBC (10^9^/L)	3.4 ± 0.9	3.4 ± 0.9	3.4 ± 0.9	0.043
RDW (%)	16.5 ± 2.8	16.4 ± 2.7	16.7 ± 2.9	0.01
Glu (mg/dL)	133.0 (104.0, 178.0)	132.0 (105.0, 178.0)	133.0 (101.0, 178.0)	0.514
BUN (mg/dL)	33.0 (20.0, 55.0)	32.0 (19.0, 53.0)	37.0 (21.0, 60.0)	0.003
Crea (mg/dL)	1.5 (0.9, 2.8)	1.5 (0.9, 2.7)	1.7 (1.0, 2.8)	0.1
PLT (10^9^/L)	185.0 (119.0, 272.0)	195.0 (126.0, 282.0)	168.5 (105.8, 247.0)	< 0.001
WBC (10^9^/L)	12.0 (7.6, 17.8)	11.6 (7.2, 16.9)	13.2 (8.5, 19.3)	< 0.001
Comorbidities, *n* (%)				
Myocardial infarct	297 (19.1)	177 (18.2)	120 (20.7)	0.226
Congestive heart failure	609 (39.2)	381 (39.2)	228 (39.3)	0.952
Chronic pulmonary disease	499 (32.1)	297 (30.5)	202 (34.8)	0.079
Diabetes	216 (13.9)	165 (17)	51 (8.8)	< 0.001
Renal disease	504 (32.5)	340 (34.9)	164 (28.3)	0.007
Malignant cancer	296 (19.1)	190 (19.5)	106 (18.3)	0.544
Severe liver disease	250 (16.1)	130 (13.4)	120 (20.7)	< 0.001
Metastatic solid tumor	148 (9.5)	72 (7.4)	76 (13.1)	< 0.001
Sofa score	4.4 ± 2.5	4.1 ± 2.2	4.8 ± 2.8	< 0.001

*HR* heart rate, *SBP* systolic blood pressure, *DBP* diastolic blood pressure, *MBP* mean blood pressure, *RR* respiratory rate, *SpO*_*2*_ oxygen saturation, *ALB* albumin, *FAR* ferritin/albumin ratio, *AG* anion gap, *HCT* hematocrit, *HGB* hemoglobin, *RBC* red blood cells, *RDW* erythrocyte distribution width, *Glu* glucose, *BUN* blood urea nitrogen, *Crea* creatinine, *PLT* platelets, *WBC *white blood cell

### The LAR is an independent risk factor for 28-day mortality in patients with sepsis

Our univariate Cox regression analysis revealed a significant association between FAR (log2) and 28-day mortality in sepsis patients (HR: 1.17, 95% CI 1.13–1.22, *P* < 0.001), as shown in Table 2. Subsequently, we included covariates with *P* < 0.001 from Table 2 in our multivariate Cox regression analysis to examine the independent effect of FAR on 28-day mortality in sepsis patients. We constructed four different multivariate Cox regression models, and the hazard ratios (HR) and 95% confidence intervals (CI) are presented in Table 3.

**Table 2:** Univariate Cox analysis of risk factors for death within 28 days in patients

Variables	Univariable COX		
	HR	95% CI	*P*
Age (years)	0.998	0.9926,1.0035	0.483
Gender (%)			
Female	Ref.		
Male	1.0048	0.8528,1.1838	0.955
Length of stay in hospital	0.9962	0.9958,0.9967	< 0.001
Length of stay in ICU	0.9986	0.9982,0.999	< 0.001
Vital signs			
HR (beats/min)	1.0036	1,1.0071	0.048
SBP (mmHg)	0.996	0.9927,0.9994	0.021
DBP (mmHg)	0.9949	0.9903,0.9995	0.029
MAP (mmHg)	0.994	0.9896,0.9984	0.008
RR (t/min)	1.02	1,1.03	0.015
SpO2 (%)	0.98	0.96,0.99	0.002
Temperature (℃)	0.93	0.86,1	0.064
ALB (g/dL)	0.79	0.7,0.9	< 0.001
Ferritin (ng/mL) (Log_2_)	1.17	1.13,1.22	< 0.001
FAR (Log_2_)	1.17	1.13,1.22	< 0.001
AG (mg/dL)	1.04	1.02,1.05	< 0.001
Bicarbonate (mg/dL)	0.98	0.96,0.99	< 0.001
Calcium (mg/dL)	0.98	0.92,1.05	0.616
Chloride, mean ± SD	0.99	0.98,1	0.038
Sodium (mEq/L)	1.0003	0.9894,1.0113	0.959
Potassium (mEq/L)	1.03	0.96,1.12	0.396
HCT (%)	0.9954	0.984,1.007	0.438
HGB (g/dL)	0.99	0.95,1.02	0.471
RBC (10^9^/L)	0.91	0.83,1	0.059
RDW (%)	1.04	1.01,1.07	0.004
Glu (mg/dL)	1	1,1	< 0.001
BUN (mg/dL)	1.0032	1.0009,1.0055	0.006
Crea (mg/dL)	1.0008	0.9643,1.0387	0.968
PLT (10^9^/L)	0.999	0.9984,0.9996	0.002
WBC (10^9^/L)	1.0073	1.0024,1.0122	0.003
Comorbidities, n(%)			
Myocardial infarct			
No	Ref.		
Yes	1.11	0.91,1.36	0.311
Congestive heart failure			
No	Ref.		
Yes	0.97	0.82,1.15	0.719
Chronic pulmonary disease			
No	Ref.		
Yes	1.13	0.95,1.34	0.169
Diabetes			
No	Ref.		
Yes	0.53	0.4,0.7	< 0.001
Renal disease			
No	Ref.		
Yes	0.76	0.63,0.91	0.002
Malignant cancer			
No	Ref.		
Yes	0.91	0.74,1.13	0.39
Severe liver disease			
No	Ref.		
Yes	1.51	1.23,1.84	< 0.001
Metastatic solid tumor			
No	Ref.		
Yes	1.6	1.25,2.03	< 0.001
Sofa score	1.1	1.07,1.13	< 0.001

*HR* heart rate, *SBP* systolic blood pressure, *DBP* diastolic blood pressure, *MBP* mean blood pressure, *RR* respiratory rate, *SpO*_*2*_ oxygen saturation, *ALB* albumin, *FAR* ferritin/albumin ratio, *AG* anion gap, *HCT* hematocrit, *HGB* hemoglobin, *RBC* red blood cells, *RDW* erythrocyte distribution width, *Glu* glucose, *BUN* blood urea nitrogen, *Crea* creatinine, *PLT* platelets, *WBC* white blood cell

**Table 3:** Multivariate Cox regression analysis of risk factors for death within 28 days in patients

Variable	Non-adjusted Model		Model I		Model II		Model III	
	HR (95%CI)	*P* value	HR (95%CI)	*P* value	HR (95%CI)	*P* value	HR (95%CI)	*P* value
Ferritin/albumin (Log_2_)	1.17 (1.13–1.22)	< 0.001	1.18 (1.13–1.23)	< 0.001	1.17 (1.11–1.24)	< 0.001	1.19(1.12–1.26)	< 0.001
Binary variable								
Ferritin/Albumin < 364.2215	Ref.		Ref.		Ref.		Ref.	
Ferritin/albumin ≥ 364.2215	2.07 (1.75–2.44)	< 0.001	2.09 (1.77–2.47)	< 0.001	1.8 (1.49–2.17)	< 0.001	1.84(1.5–2.25)	< 0.001

Model I = Adjusted for age + gender. Model II = Model I + length of stay in hospital + length of stay in ICU + ALB + Ferritin + AG + Bicarbonate + Glu + Diabetes + Severe liver disease + Metastatic solid tumor + Sofa score. Model III = Model II + HR + SBP + DBP + MBP + RR + Spo2 + Temperature + Calcium + Chloride + Sodium + Potassium + HCT + HGB + RBC + RDW + BUN + Crea + PLT + WBC + Myocardial infarct + Congestive heart failure + Chronic pulmonary disease + Renal disease + Malignant cancer

*HR* heart rate, *SBP* systolic blood pressure, *DBP* diastolic blood pressure, *MBP* mean blood pressure, *RR* respiratory rate, *SpO*_*2*_ oxygen saturation, *ALB* albumin, *FAR* ferritin/albumin ratio, *AG* anion gap, *HCT* hematocrit, *HGB* hemoglobin, *RBC* red blood cells, *RDW* erythrocyte distribution width, *Glu* glucose, *BUN* blood urea nitrogen, *Crea* creatinine, *PLT* platelets, *WBC* white blood cell

**Fig. 2:**
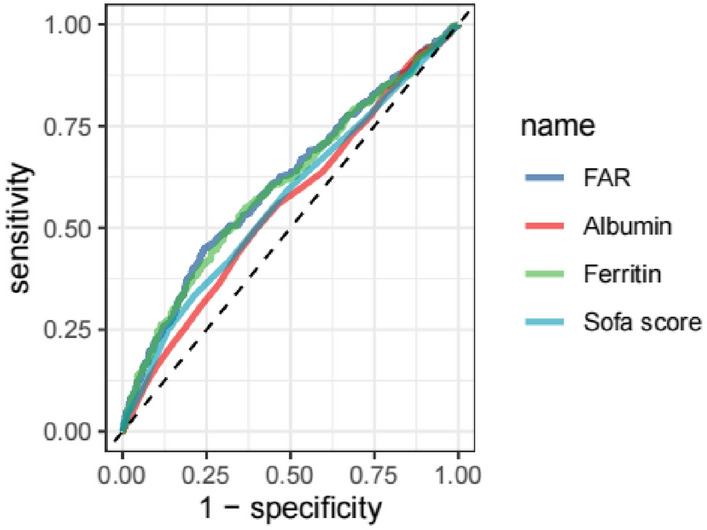
ROC curve analysis

In the minimally adjusted model (Model I), we observed a persistent significant association between FAR and 28-day mortality in sepsis patients (HR: 1.18, 95% CI 1.13–1.23, *P* < 0.001). Even after full adjustment for covariates (Model III), FAR was still an independent risk factor. To further assess the robustness of our findings, we converted the continuous variable FAR to a dichotomous variable based on the FAR threshold derived from the ROC curve in Fig. 2. Using a cutoff point of FAR < 364.2215 as the reference baseline, we obtained similar conclusions, indicating that FAR is an independent risk factor for 28-day death in sepsis patients.

### ROC curve analysis and survival curve analysis

We performed an ROC curve analysis on four indicators, namely, FAR, ferritin, albumin, and SOFA score, to predict the 28-day mortality rate in sepsis patients. Table 4 shows the data from Fig. 2. The results revealed that FAR had a larger AUC (61.0141%) than ferritin (60.4778%), albumin (55.5618%), and even the SOFA score (56.9719%), indicating a significant predictive advantage of FAR.

**Table 4:** Information of ROC curves in Fig. 2

Variables	AUC (%)	95% CI (%)	Threshold	Sensitivity	Specificity
FAR	61.0141	58.0655–63.9627	364.2215	0.4483	0.7585
Ferritin	60.4778	57.5161–63.4396	821.5	0.4897	0.6968
Albumin	55.5618	52.5937–58.5299	2.75	0.4966	0.6023
Sofa score	56.9719	54.0308–59.9129	5.5	0.2158	0.6638

**Fig. 3:**
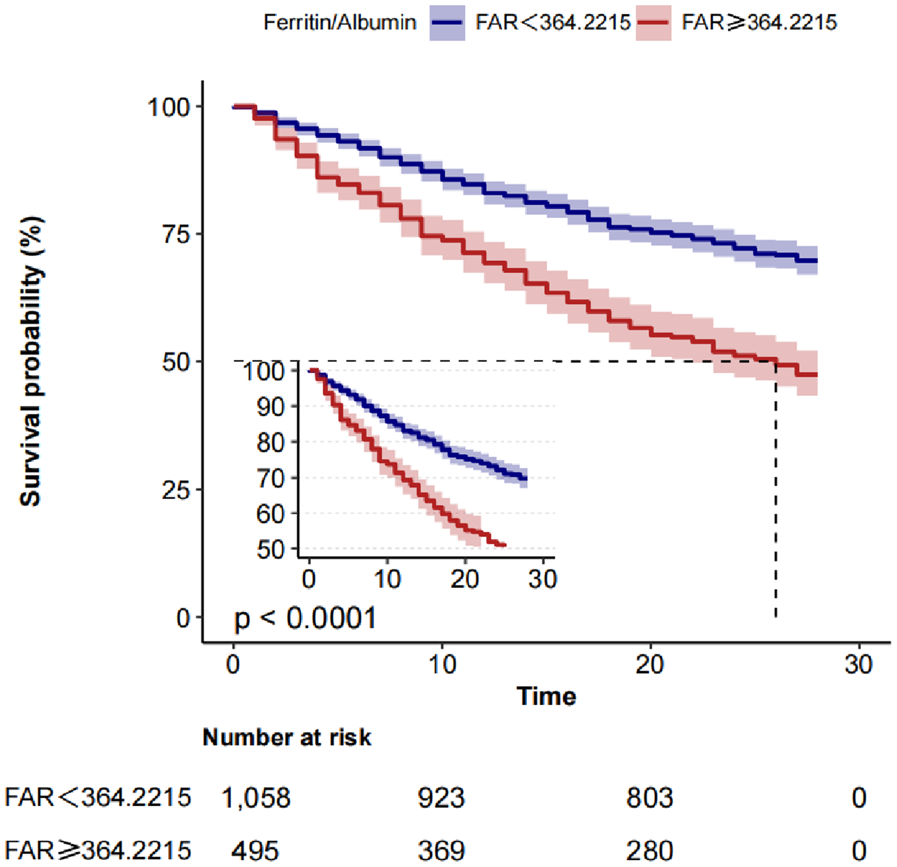
Kaplan–Meier survival analysis curves for 28-day mortality in patients with sepsis

Furthermore, we have determined the optimal threshold for FAR to be 364.2215, enabling the categorization of sepsis patients into high FAR group (FAR ≥ 364.2215) and low FAR group (FAR < 364.2215). Subsequently, as shown in Fig. 3, survival curves were constructed to compare the survival rates between the two groups of patients, revealing a significantly higher mortality rate in the high FAR group compared to the low FAR group (*P* < 0.001).

### Subgroup analysis

**Fig. 4:**
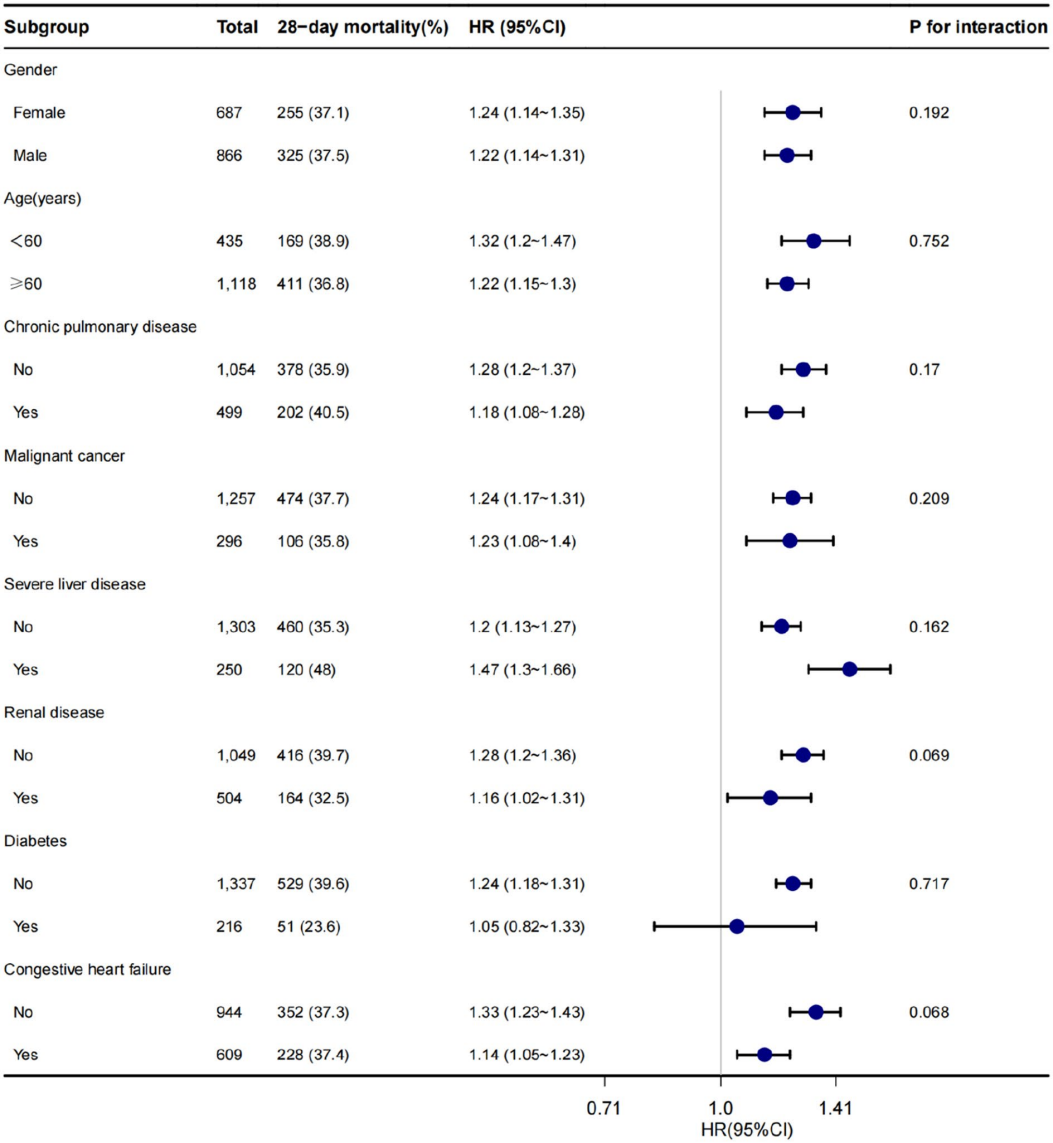
Subgroup analysis forest plot depicting the relationship between 28-day mortality rate and FAR in sepsis patients

The forest plot (Fig. 4) demonstrated that there was no observed interaction between FAR and any of the subgroups when stratified by variables, such as age, gender, diabetes, congestive heart failure, liver disease, kidney disease, chronic pulmonary disease, and malignant cancer (*P* > 0.05). These findings strongly suggest that, regardless of these demographic and clinical factors, FAR remains an independent risk factor for 28-day mortality in sepsis patients.

## Discussion

The findings of this retrospective study strongly indicate that FAR is a significant independent factor associated with a 28-day all-cause mortality in sepsis patients and serves as a reliable predictor for predicting 28-day mortality in sepsis patients. The analysis of the ROC curve demonstrates that FAR exhibits a higher AUC value (61.0141%) compared to ferritin (60.4778%), albumin (55.5618%), and SOFA score (56.9719%). Moreover, the comprehensive analysis, including multifactor COX regression and Kaplan–Meier survival analysis, reveals a positive correlation between FAR and the 28-day mortality rate among sepsis patients. Notably, as FAR increases, the risk of 28-day mortality decreases. The findings are further reinforced by the subgroup analysis, ensuring the robustness and validity of our results.

In recent years, an increasing number of researchers have found that inflammatory biomarkers, such as ALB [17], ferritin [6], Albumin/Fibrinogen [18, 19], hematocrit [20], platelets [21], Red blood cell distribution width [22], systemic immune-inflammation index [23], Prognostic nutrition index [24], and urea nitrogen/albumin [25], etc. FAR holds potential as a prognostic indicator for predicting the outcome of sepsis patients. FAR has been widely employed as a novel predictive factor for COVID-19 [16], exhibiting excellent prognostic accuracy for mortality rates. However, studies specifically exploring the application of FAR in predicting the prognosis of sepsis patients have yet to be reported.

Ferritin plays a vital role in iron storage and regulation, transportation of iron ions, immune modulation, and antioxidation within the body. Our research findings strongly support the close association between elevated serum ferritin levels and poor prognosis in sepsis patients, consistent with the study conducted by Liudang He et al. [6]. In addition, the study by Yi-Peng Fang et al. [26] revealed a significant correlation between high serum ferritin levels and increased in-hospital mortality rate in sepsis patients, establishing it as an independent prognostic indicator for predicting mortality in septic patients. However, it is important to consider that factors, such as inflammation, liver and kidney function, as well as dietary and nutritional status, can also influence serum ferritin levels. Thus, relying solely on ferritin as a prognostic marker may have limitations and lack reliability to predict patient outcomes.

The pathogenesis of sepsis is closely tied to the inflammatory response, which triggers vasodilation, thrombus formation, cell apoptosis, and tissue damage. Serum albumin, being a negative acute-phase reactant protein, is correlated with the severity of infection [27, 28]. During sepsis, the inflammatory response induced by infection influences the synthesis and degradation of albumin, resulting in reduced levels of albumin in the plasma and causing hypoalbuminemia [29, 30]. Recent research indicates that hypoalbuminemia in sepsis patients is not primarily caused by suppressed liver synthesis, but rather by enhanced systemic clearance [31]. Studies indicate that serum albumin is an independent prognostic risk factor for sepsis patients [13, 17]. Nevertheless, it is important to consider that serum albumin levels can be influenced by various factors, including nutritional status, hepatic and renal function, chronic inflammatory conditions, and other underlying medical conditions of the patients. Therefore, this study aims to evaluate prognostic risks in sepsis patients more comprehensively by analyzing the relationship between the ferritin-to-albumin ratio and the 28-day mortality rate, thereby mitigating the influence of individual factors on sepsis prognosis.

Although there have been numerous studies investigating the relationship between ferritin and albumin and various diseases, the association between the ferritin-to-albumin ratio (FAR) and sepsis remains unexplored. However, research indicates that elevated ferritin levels and decreased albumin levels serve as important markers for mortality and prognosis in sepsis patients. Based on this information, we propose that FAR may hold greater significance in the context of sepsis compared to individual measurements of ferritin and albumin. In our study, we discovered a positive correlation between FAR and the 28-day mortality rate among sepsis patients, suggesting that higher FAR levels are associated with an increased risk of death in this population. These findings align with the conclusions drawn by Öztürk Taşkin et al. regarding the prognostic value of FAR in predicting mortality in COVID-19 patients [16]. Furthermore, ROC curves demonstrated that FAR exhibits good predictive capabilities with regard to the 28-day mortality rate among sepsis patients.

Our study has several advantages. First, the data used in our study is obtained from the MIMIC-IV database, ensuring the authenticity and reliability of the data. Second, this is a retrospective study, where we employed various statistical methods such as multiple-factor Cox regression analysis and subgroup analysis to control for potential confounding factors in the study. In addition, we selected the levels of ferritin and albumin measured for the first time after patient admission, excluding the interference of subsequent treatments, thereby enhancing the accuracy of the results. Finally, our study is the first to explore the relationship between FAR and the 28-day mortality rate in sepsis patients. Moreover, the convenient and cost-effective acquisition of serum ferritin and albumin levels further enhances the feasibility of this approach.

Certainly, Our study does have certain limitations. First, One significant limitation of our study is that we solely relied on the initial measurement of FAR after admission, failing to continuously track its fluctuations throughout the entire duration of sepsis. As a result, we were unable to assess the potential impact of dynamic changes in FAR on the prognosis of sepsis patients. Second, the data used in our study was obtained from the MIMIC-IV public database. As a retrospective study, despite our best efforts to control for confounding factors, it is still challenging to completely avoid the influence of unknown confounders. Finally, the measurement of laboratory indicators was not strictly standardized, and different hospitals may use different instruments, leading to measurement deviations in the results.

## Conclusion

FAR, which has the potential to serve as an indicator for predicting the prognosis of sepsis patients, shows a correlation with the 28-day mortality rate. A higher FAR value is associated with an elevated risk of mortality within 28 days among sepsis patients. However, to comprehensively validate the relationship between FAR and sepsis prognosis, a large-scale study involving multiple medical centers is necessary.

## Data Availability

The data sets analyzed in this study are available upon reasonable request from the corresponding author.

## References

[1] Singer M, Deutschman CS, Seymour CW, Shankar-Hari M, Annane D, Bauer M, Bellomo R, Bernard GR, Chiche JD, Coopersmith CM (2016). The third international consensus definitions for sepsis and septic shock (Sepsis-3). JAMA.

[2] Bauer M, Gerlach H, Vogelmann T, Preissing F, Stiefel J, Adam D (2020). Mortality in sepsis and septic shock in Europe, North America and Australia between 2009 and 2019—results from a systematic review and meta-analysis. Crit Care.

[3] Fleischmann-Struzek C, Mellhammar L, Rose N, Cassini A, Rudd KE, Schlattmann P, Allegranzi B, Reinhart K (2020). Incidence and mortality of hospital- and ICU-treated sepsis: results from an updated and expanded systematic review and meta-analysis. Intensive Care Med.

[4] Cecconi M, Evans L, Levy M, Rhodes A (2018). Sepsis and septic shock. Lancet.

[5] Williams V, Menon N, Bhatia P, Biswal M, Sreedharanunni S, Rawat A, Jayashree M, Nallasamy K (2020). Serum ferritin predicts neither organ dysfunction nor mortality in pediatric sepsis due to tropical infections. Front Pediatr.

[6] He L, Guo C, Su Y, Ding N (2023). The relationship between serum ferritin level and clinical outcomes in sepsis based on a large public database. Sci Rep.

[7] Shaikh GN, Ramamoorthy JG, Parameswaran N, Senthilkumar GP (2022). Serum ferritin for predicting outcome in children with severe sepsis in the pediatric intensive care unit. Indian Pediatr.

[8] Tran TN, Tran HD, Tran-Huu TT, Tran DM, Tran QN (2022). A cross-sectional study of serum ferritin levels in Vietnamese adults with metabolic syndrome. Diabetes Metab Syndr Obes.

[9] Meier JA, Bokemeyer A, Cordes F, Fuhrmann V, Schmidt H, Husing-Kabar A, Kabar I (2020). Serum levels of ferritin and transferrin serve as prognostic factors for mortality and survival in patients with end-stage liver disease: a propensity score-matched cohort study. United European Gastroenterol J.

[10] Zhu Z, Yin J, Dawsey SM, Liu B, Freedman ND, Yin L, Taylor PR, Cui J, Fan J, Liu Y (2021). Association between serum ferritin, incident primary liver cancer, and chronic liver disease mortality in the Linxian Nutrition Intervention Trials: a nested case–control study. J Gastroenterol Hepatol.

[11] Qeadan F, Tingey B, Gu LY, Packard AH, Erdei E, Saeed AI (2021). Prognostic values of serum ferritin and d-dimer trajectory in patients with COVID-19. Viruses.

[12] Shin J, Hwang SY, Jo IJ, Kim WY, Ryoo SM, Kang GH, Kim K, Jo YH, Chung SP, Joo YS (2018). Prognostic value of the lactate/albumin ratio for predicting 28-day mortality in critically ILL sepsis patients. Shock.

[13] Arnau-Barres I, Guerri-Fernandez R, Luque S, Sorli L, Vazquez O, Miralles R (2019). Serum albumin is a strong predictor of sepsis outcome in elderly patients. Eur J Clin Microbiol Infect Dis.

[14] Onal C, Gultekin M, Yavas G, Oymak E, Yuce SS, Guler OC, Yigit E, Yildiz F (2022). The impact of serum albumin-to-alkaline phosphatase ratio in cervical cancer patients treated with definitive chemoradiotherapy. J Obstet Gynaecol.

[15] Sun J, Su H, Lou Y, Wang M (2021). Association between serum albumin level and all-cause mortality in patients with chronic kidney disease: a retrospective cohort study. Am J Med Sci.

[16] Taskin O, Yilmaz A, Soylu VG, Demir U, Catan IF (2023). Ferritin/albumin ratio could be a new indicator of COVID-19 disease mortality. J Infect Dev Countries.

[17] Cao Y, Su Y, Guo C, He L, Ding N (2023). Albumin level is associated with short-term and long-term outcomes in sepsis patients admitted in the ICU: a large public database retrospective research. Clin Epidemiol.

[18] Tai H, Zhu Z, Mei H, Sun W, Zhang W (2020). Albumin-to-fibrinogen ratio independently predicts 28-day mortality in patients with peritonitis-induced sepsis. Mediators Inflamm.

[19] Li S, Shen Y, Chang B, Wang N (2022). Prognostic value of albumin-to-fibrinogen ratio for 28-day mortality among patients with sepsis from various infection sites. Mediators Inflamm.

[20] Luo M, Chen Y, Cheng Y, Li N, Qing H (2022). Association between hematocrit and the 30-day mortality of patients with sepsis: a retrospective analysis based on the large-scale clinical database MIMIC-IV. PLoS ONE.

[21] Zhao L, Zhao L, Wang YY, Yang F, Chen Z, Yu Q, Shi H, Huang S, Zhao X, Xiu L (2020). Platelets as a prognostic marker for sepsis: a cohort study from the MIMIC-III database. Medicine (Baltimore).

[22] Xu W, Huo J, Chen G, Yang K, Huang Z, Peng L, Xu J, Jiang J (2022). Association between red blood cell distribution width to albumin ratio and prognosis of patients with sepsis: a retrospective cohort study. Front Nutr.

[23] Jiang D, Bian T, Shen Y, Huang Z. Association between admission systemic immune-inflammation index and mortality in critically ill patients with sepsis: a retrospective cohort study based on MIMIC-IV database. Clin Exp Med. 2023:1–10. 10.1007/s10238-023-01029-w PMC10022570 36930382

[24] Wu H, Zhou C, Kong W, Zhang Y, Pan D (2022). Prognostic nutrition index is associated with the all-cause mortality in sepsis patients: a retrospective cohort study. J Clin Lab Anal.

[25] Min J, Lu J, Zhong L, Yuan M, Xu Y (2022). The correlation study between blood urea nitrogen to serum albumin ratio and prognosis of patients with sepsis during hospitalization. BMC Anesthesiol.

[26] Fang YP, Zhang HJ, Guo Z, Ren CH, Zhang YF, Liu Q, Wang Z, Zhang X (2022). Effect of serum ferritin on the prognosis of patients with sepsis: data from the MIMIC-IV database. Emerg Med Int.

[27] Akirov A, Masri-Iraqi H, Atamna A, Shimon I (2017). Low albumin levels are associated with mortality risk in hospitalized patients. Am J Med.

[28] Atrash AK, de Vasconcellos K (2020). Low albumin levels are associated with mortality in the critically ill: a retrospective observational study in a multidisciplinary intensive care unit. South Afr J Crit Care.

[29] Nasa P, Juneja D, Singh O (2012). Severe sepsis and septic shock in the elderly: an overview. World J Crit Care Med.

[30] Churpek MM, Snyder A, Han X, Sokol S, Pettit N, Howell MD, Edelson DP (2017). Quick sepsis-related organ failure assessment, systemic inflammatory response syndrome, and early warning scores for detecting clinical deterioration in infected patients outside the intensive care unit. Am J Respir Crit Care Med.

[31] Omiya K, Sato H, Sato T, Wykes L, Hong M, Hatzakorzian R, Kristof AS, Schricker T (2021). Albumin and fibrinogen kinetics in sepsis: a prospective observational study. Crit Care.

